# Association of tumour-associated macrophages with cancer cell EMT, invasion, and metastasis of Kazakh oesophageal squamous cell cancer

**DOI:** 10.1186/s13000-019-0834-0

**Published:** 2019-06-12

**Authors:** Jihong Liu, Chunxiao Li, Liyan Zhang, Kai Liu, Xianli Jiang, Xueli Wang, Lan Yang, Weihua Liang, Kunping Liu, Jianming Hu, Feng Li

**Affiliations:** 1 Department of Anatomic and Surgical Pathology, and Key Laboratory of Xinjiang Endemic and Ethnic Diseses, The First Affiliated Hospital, Shihezi University School of Medicine, Xinjiang, 832002 People’s Republic of China; 2 Department of Pathology and Medical Research Center, Beijing Chao Yang Hospital, Capital Medical University, Beijing, 100020 People’s Republic of China; 3 Department of Pathology, The Sixth Affiliated Hospital of Guangzhou Medical University, Qingyuan People’s Hospital, Qingyuan, Guangdong 511518 People’s Republic of China; 4 Department of Neurology, Beijing Friendship Hospital,Capital Medical University, Beijing, 100020 People’s Republic of China

**Keywords:** Oesophageal squamous cell carcinoma, Tumour-associated macrophages, Kazakh, Epithelial-mesenchymal transition, Migration and invasion

## Abstract

**Background:**

Tumour-associated macrophages (TAMs) play an important role in the growth, progression, and metastasis of tumours. Epithelial-mesenchymal transition (EMT) is a mechanism for tumour invasion and metastasis. In this study, we aimed to determine whether TAMs can induce EMT for the invasion and metastasis of Kazakh oesophageal squamous cell cancer (ESCC).

**Methods:**

CD163 was used as a marker for TAMs, and the density of TAMs in tumour nest and surrounding stroma was quantified using immunohistochemistry (IHC). IHC staining was used to evaluate the expression of E-cadherin (epithelial marker) and vimentin (mesenchymal marker) in Kazakh ESCC and cancer-adjacent normal tissues (CANs). Additionally, 6-well transwell plates (0.4 μm) were used to establish the co-culture system of ESCC (EC109 or EC9706) cells and macrophages. Real-time quantitative polymerase chain reaction (qPCR) and western blot experiments were used to determine whether ESCC cells undergo EMT transformation after co-culture with macrophages. Transwell assays were used to detect the migration and invasion of the ESCC cells.

**Results:**

The distribution of CD163-positive TAMs in cancer tissues was closely related to EMT in Kazakh ESCC. The expression of vimentin in the ESCC cells was significantly upregulated, the expression of E-cadherin was significantly downregulated, and the invasion and migration of the ESCC cells were significantly enhanced after tumour-associated macrophages were added to the co-culture.

**Conclusions:**

Tumour-associated macrophages promote EMT in ESCC, which may be one of the important factors involved in the invasion and progression of Kazakh ESCC.

## Background

Oesophageal cancer (EC) is the eighth most common malignancy in the world and is the sixth most common cause of cancer deaths [1]. The incidence and mortality rates of Kazakh EC in northwest Xinjiang are higher than the average levels in China [2]. Early invasion and metastasis may be the main reasons for poor prognosis of Kazakh EC.

There is growing evidence that the tumour microenvironment is important for tumour progression and metastasis [3–5]. Macrophages are important mesenchymal cells in the tumour microenvironment that play an important role in promoting tumour progression, angiogenesis, and metastasis [6]. Macrophages possess plasticity and are classified into different subtypes in different environments, namely, M1 classically activated macrophages, and M2 alternatively activated macrophages [6]. Macrophages can differentiate into M1 macrophages in the presence of lipopolysaccharide (LPS) and interferon gamma (IFNγ), release a series of cytokines, result in the low expression of interleukin (IL-10) and high expression of IL-12, and inhibit and kill cancer cells. Macrophages can differentiate into the M2 subtype in the presence of IL-4 and IL-13, low expression of IL-12, and high expression of IL-10, which plays an important role in inhibiting inflammation and promoting tumour growth and immunosuppression [7]. Macrophages present in tumours (tumour-associated macrophages or TAMs) exhibit M2 phenotype and are capable of expressing CD68 and CD163 [8]. Recent studies have shown that CD163-positive macrophages are associated with histological grade and poor prognosis of tumours [9]. TAM infiltration density is associated with recurrent and poor prognosis in a variety of tumours such as breast cancer [10], leiomyosarcoma [11], and oesophageal cancer [6]. However, the underlying mechanisms of TAMs in the invasion and metastasis of EC are unclear.

Currently, epithelial-mesenchymal transition (EMT) is considered a mechanism of tumour invasion and metastasis [12]. EMT refers to the conversion of epithelial cells into mesenchymal cells under certain conditions [13]. The cells are rounded into a spindle shape and become motile and invasive [14]. A series of evidence suggests that TAMs can promote invasion and metastasis of breast cancer [15], lung cancer [16], and hepatocellular carcinoma [17] through EMT. However, the role of EMT in TAM-promoted invasion and metastasis of ESCC is not yet known.

Therefore, CD163 was used as a marker of M2 TAMs, E-cadherin and vimentin were used as epithelial and mesenchymal markers, respectively, and the co-culture system was established, to explore whether TAMs could induce EMT for the invasion and metastasis of Kazakh ESCCs.

## Methods

### Patients and specimens

Surgically resected and paraffin-embedded human tissues were collected from the Department of Pathology at Yili Friendship Hospital in Xinjiang, China, between 2008 and 2014. One hundred cases of Kazakh ESCC and 100 cases of Kazakh cancer-adjacent normal (CAN) tissues were collected for this study(some of cases overlaps with previous study [18]). Of all patients, 62 were males and 38 females, with ages ranging from 33 to 76 years and a mean age of 58 years. All patients were diagnosed with ESCC were confirmed by two pathologists according to the World Health Organization histological tumour classification criteria. In this study, there were 26 patients with well differentiated, 47 patients with moderately differentiated, and 27 patients with poorly differentiated ESCCs. From these, 29 and 71 patients exhibited invasive depths of T1-T2 and T3-T4, respectively. There were 45 patients with lymph node metastasis, 68 patients with lymphatic metastasis, 65 patients in clinical stages I–II, and 35 patients in clinical stages III–IV. None of the patients accepted chemotherapy or radiation therapy before surgery.

### Immunohistochemistry (IHC)

For IHC, anti-CD163 (clone 10D6, Zhongshan Goldenbridge Biotechnology Co., LTD., Beijing, China) was diluted 1:100. Both anti-E-cadherin and anti-vimentin antibodies were purchased from Cell Signaling Technology (Danvers, MA, USA) and diluted 1:1000. The sections were thoroughly washed with PBS and the primary antibody binding was visualized using the DAKO Envision kit (DAKO, Glostrup, Denmark) according to the manufacturer’s instructions. IHC was performed as reported previously [19].

### Immunoreactivity evaluation

CD163-positive macrophages were analysed as described previously [19]. Tumour nest and stroma areas were defined, and CD163-positive macrophages were counted under high-power fields (HPFs) (400×) by two pathologists who were blinded to the clinical patient data. When cell counts differed by more than 10 cells per HPF, the cells would be counted again a week later until the recorded differences were below 10 counts. The mean number of macrophages per HPF across five hot spots for every sample (tumour islet and tumour stroma) was defined as the M2 macrophage density.

E-cadherin and vimentin were positively expressed on the membrane or cytoplasm of the cell, which were defined as a yellow-brown colour, following the manufacturer’s guidelines. IHC-stained slides were scored as positive or negative by the percentage and intensity of positive cells. The scoring percentages of positively stained cells were as follows: 0, < 5%; 1, 6–25%; 2, 26–50%; 3, 51–75%; and 4, 76–100%. The staining intensity scoring was as follows: 0, absent; 1, weak; 2, moderate; and 3, strong. A final score was based on the multiplication of both scores from individual slides [19], where 0–1 was negative (−), 2–3 was weakly positive (1+), 4–6 was moderately positive (2+), and 8–12 was strongly positive (3+).

### Cell culture

Human ESCC cell lines EC109 and EC9706 and the human acute monocytic leukaemia cell line THP-1 were purchased from the American Type Culture Collection (ATCC; Manassas, VA, USA). These cells were cultured in RPMI-1640 medium (Invitrogen, Carlsbad, CA, USA) containing 10% foetal bovine serum (FBS; Invitrogen, Carlsbad, CA, USA) and 1% penicillin/streptomycin solution (Invitrogen, Carlsbad, CA, USA). THP-1 cells were additionally incubated with 0.05 mM β-mercaptoethanol. All cells were incubated at 37 °C in a humidified cell culture incubator with 5% CO_2_. THP-1 cells were treated with Phorbol-12-myristate-13-acetate (PMA, 10 ng/mL; Sigma, USA) for 36 h to induce THP-1 to macrophages, then macrophages and EC109 or EC9706 cells (4 × 10^4^ cells) were separately cultured in the upper and lower chamber of 6-well transwell plates (Corning Inc., Corning, NY) for 48 h without contact, the supernatant was collected and filtered, which was co-cultured conditioned medium (CM). At the same time, ESCC cells cultured in the DMEM for 48 h, the supernatant was collected and filtered, which was routine medium (RM) as control. Finally, the co-cultured conditioned medium (CM) and RM was collected for subsequent studies.

### Quantitative polymerase chain reaction (qPCR)

Total RNA was extracted using TRIzol reagent (Invitrogen) according to the manufacturer’s instructions, and mRNA was reverse-transcribed into cDNA by using the TIANScript RT Kit (TIANGEN, China) at 37 °C for 15 min and 85 °C for 5 s, according to the manufacturer’s protocol. qPCR was performed to evaluate the gene expression using QuantiFast SYBR Green PCR (QIAGEN) with a 7500 fast Real-Time PCR System (Applied Biosystems, Foster City, CA, USA) under the following conditions: 40 cycles at 95 °C for 30 s, 95 °C for 5 s, and 60 °C for 31 s. The primers used were previously published [20].

### Western blotting

Cells were washed 3 times with ice-cold phosphate buffer saline (PBS) and were lysed by lysis buffer on ice for 30 min, followed by centrifugation at 12,000 rpm for 10 min at 4 °C to remove cell debris. Protein samples were mixed with loading buffer, heated at 100 °C for 10 min, and separated on 10% sodium dodecyl sulphate- polyacrylamide gel electrophoresis (SDS-PAGE) gels (Bio-Rad, California, USA). Proteins were then transferred to polyvinyl difluoride membranes (Solarbio Systems, Beijing, China). The blots were incubated with primary antibodies overnight at 4 °C with rabbit anti-E-cadherin (1:400) (Cell Signaling Technology, Danvers, MA, USA) and rabbit anti-vimentin (1:400) (Cell Signaling Technology, Danvers, MA, USA). This procedure was followed by incubation with an anti-rabbit HRP-labelled secondary antibody (1:20,000) for 2 h at 25 °C, followed by detection with an Enhanced-Chemi-Luminescence (ECL) reagent kit (Thermo Systems, Massachusetts, USA). Immunoblots for β-actin served as a loading control. All experiments were repeated in triplicate.

### Cell migration and invasion assays

Cells (4 × 10^4^) were diluted in 200 μL serum-free medium and then added to the top chambers of 24-well plates (Corning, Corning, NY, USA); 600 μL of 20% FBS was added to the lower chamber to induce cell migration. Each chamber was coated freshly with 50 μg Matrigel (Becton, Dickinson and Company, Franklin Lakes, NJ, USA) before performing the invasion assay. After 24 h, the cells were fixed with 4% paraformaldehyde and then stained with 0.1% crystal violet. The number of migrated and invasive cells was calculated in five random fields per well using an inverted light microscope.

### Statistical analysis

Data were analysed using the SPSS 17.0 software package. Two sets of measurement data were compared using Independent-Samples *t* test and χ2 test. Comparisons between multiple groups were performed using Partial correlation analysis. *P* values less than 0.05 were considered statistically significant. P values were corrected with Bonferroni Correction for multiple testing.

## Results

### Distribution of TAMs in Kazakh ESCC

**Fig. 1:**
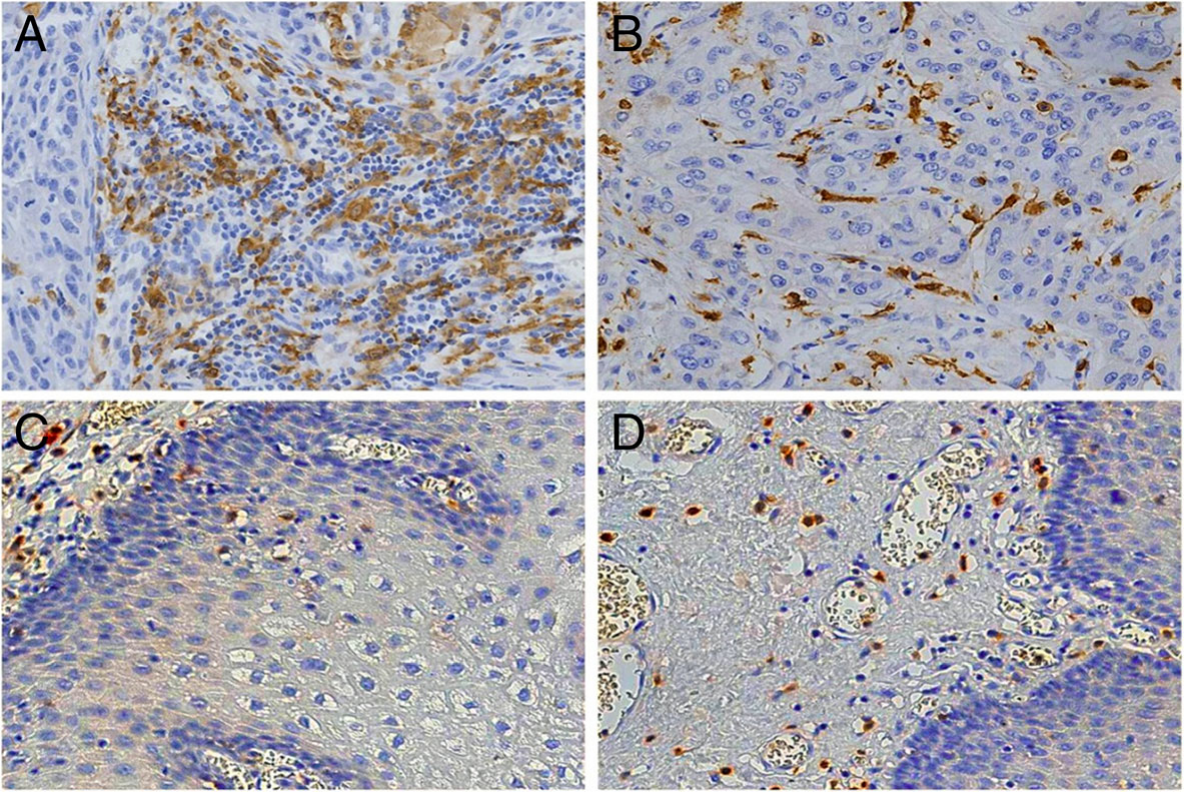
Distribution of CD163-positive M2 macrophages in Kazakh ESCC and CAN tissues. (**a**) and (**b**) showed the distribution of M2 macrophages in ESCC tumour stromal and islet, respectively. CD163 revealed diffuse staining of membranes and cytoplasm of M2 macrophages and showed the high density of M2 macrophages located in ESCC tissues (especially in tumour stroma). (**c**) and (**d**) showed the distribution of TAMs in CAN stroma and epithelia, respectively. A small number of CD163-positive M2 macrophages appear in CAN tissues

In this study, we used CD163 as a marker of TAMs to assess their distribution in the Kazakh ESCC patients. CD163 was localized in the cell membrane or in the cytoplasm of macrophages. The TAMs mainly localized in the stroma of cancerous tissues, and a few localized in the cancerous nest (Fig. 1). The number of TAMs in ESCC was significantly higher in the interstitial tissues (58 per HPF) and in the nest (13 per HPF) than in the CAN stroma (22 per HPF) and epithelium (2 per HPF), and the difference was statistically significant (*p* < 0.05) (Table 1).

**Table 1:** The distribution of CD163-positive macrophages in Kazakh ESCCs and CANs

Groups	Cases(N)	Islets (mean ± SD)	*P*	Stroma (mean ± SD)	*P*
ESCCs	100	13.43 ± 9.22	<0.001	57.96 ± 26.53	<0.001
CANs	100	1.82 ± 1.71		22.23 ± 9.85	

### Expression of EMT-related proteins in Kazakh ESCC and their correlation with clinicopathological parameters

**Fig. 2:**
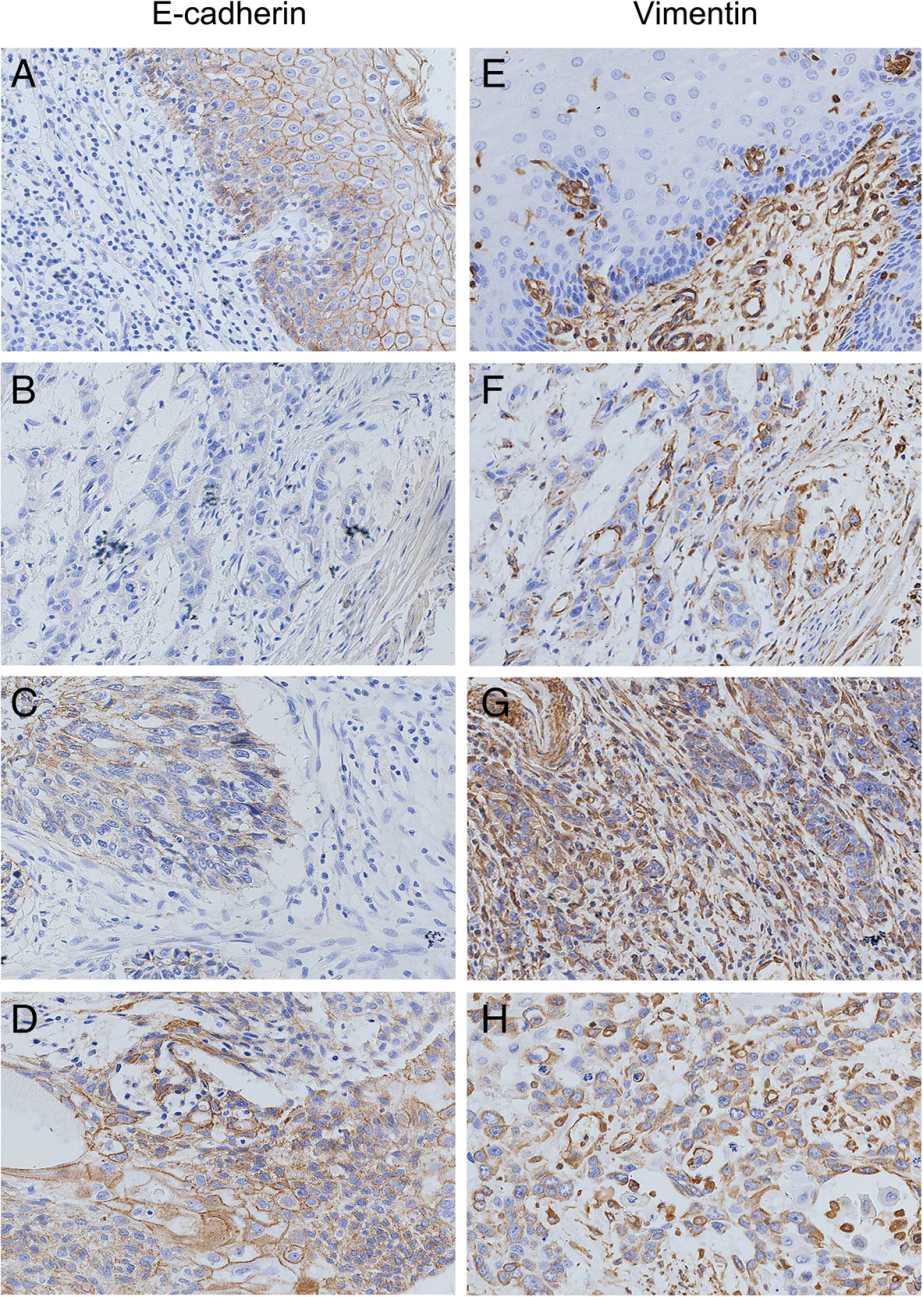
Detection of E-cadherin and Vimentin expression in Kazakh ESCC and CANs by IHC E-cadherin and vimentin staining was localized predominantly in the cytomembrane and (or) cytoplasm. (**a**) Positive E-cadherin staining is shown in CAN tissues (scored as 3). (**b**) Negative E-cadherin staining is shown in CAN tissues (scored as 0). (**c**) and (**d**) show weak and moderate E-cadherin staining in Kazakh ESCC tissues, respectively (scored as 1 and 2, respectively). (**e**) Negative vimentin staining is shown in CAN tissues (scored as 0). (**f**) Weak vimentin staining is shown in ESCC tissues (scored as 1). (**g**) and (**h**) show moderate and strong vimentin staining in Kazakh ESCC tissues, respectively (scored as 2 and 3, respectively)

We used E-cadherin and vimentin as markers for the epithelium and mesenchyme, respectively, to detect EMT in Kazakh ESCC. We found that E-cadherin predominantly localized in the cell membrane and vimentin localized in the cell membrane or cytoplasm (Fig. 2). The positive expression of E-cadherin in ESCC tumour tissues was 58% (58/100), which was significantly lower than that in CANs tissues (96%, *p* < 0.05) (Table 2). While the expression of vimentin in ESCC tumour tissues was 47% (47/100), and none of the normal tissues expressed vimentin in CAN tissues (0%), (Table 2). We further examined the correlation of EMT-related protein expression and the clinicopathological characteristics as shown in Table 3. Despite no significant correlations with sex and age, we observed a significant negative correlation between E-cadherin expression and tumour differentiation, clinical stage, depth of invasion, lymph node metastasis, and vascular invasion (*p* < 0.05), Moreover, we found that the expression of E-cadherin was negatively correlated with tumour differentiation, especially between moderate differentiation and poor differentiation (*p* < 0.0125). However, we found that the expression of vimentin was positively correlated with gender, depth of invasion, clinical stage, lymph node metastasis, and vascular invasion (*p* < 0.05). No positive correlations were found between other clinicopathological parameters and vimentin expression.

**Table 2:** The expression of E-cadherin and Vimentin in Kazakh esophageal ESCC CANs

Groups	Cases	E-cadherin expression		X^2^	*P*	Vimentin expression		*X* ^*2*^	*P*
		Low	high			low	high		
		0/1 + (%)	2+/3 + (%)			0/1 + (%)	2+/3 + (%)		
ESCCs	100	42(42%)	58(58%)	40.768	<0.001	53(53%)	47(47%)	61.438	<0.001
CANs	100	4(4%)	96(96%)			100(100%)	0(0%)

**Table 3:** Correlation between expression of EMT and clinicopathological parameters in Kazakh ESCCs

Variable	Cases	E-cadherin low expression 0/1 + (%)	E-cadherin high expression 2+/3 + (%)	X^2^	*P*	Vimentin low expression 0/1 + (%)	Vimentin high expression 2+/3 + (%)	X^2^	*P*
Age(y)									
≤58	58	29(50%)	29(50%)	3.628	0.057	30(51.7%)	28(48.3%)	0.090	0.764
> 59	42	13(31%)	29(69%)			23(54.8%)	19(45.2%)		
Gender									
Male	62	30(48.4%)	32(51.6%)	2.732	0.098	26(41.9%)	36(58.1%)	8.109	0.005*
Female	38	12(31.6%)	26(68.4%)			27(71.1%)	11(28.9%)		
Differentiation									
High	26	9(34.6%)	17(65.4%)	0.055^a^	0.814^a^	13(50%)	13(50%)		
Moderate	47	15(31.9%)	32(68.1%)	8.382^b^	0.004^b#^	30(63.8%)	17(36.2%)	5.069	0.079
Poor	27	18(66.7%)	9(33.3%)	5.445^c^	0.020^c^	1(37%)	2(63%)		
Depth of invasion									
T1-T2	29	3(10.3%)	26(89.7%)	16.802	<0.001*	25(86.2%)	4(13.8%)	18.081	< 0.001*
T3-T4	71	39(54.9%)	32(45.1%)			28(39.4%)	43(60.6%)		
Clinical stage									
I-II	65	19(29.2%)	46(70.8%)	12.431	<0.001*	45(69.2%)	20(30.8%)	19.640	< 0.001*
III-IV	35	23(65.7%)	12(34.3%)			8(22.9%)	27(77.1%)		
Lymph node metastasis									
pN-	55	18(32.7%)	37(67.3%)	4.314	0.038*	37(67.3%)	18(32.7%)	9.995	0.002*
pN+	45	24(53.3%)	21(46.7%)			16(35.6%)	29(64.4%)		
Lymphatic metastasis									
pN-	32	7(21.9%)	25(78.1%)	7.824	0.005*	23(71.9%)	9(28.1%)	6.730	0.009*
pN+	68	35(51.5%)	33(48.5%)			30(44.1%)	43(55.9%)		

*p < 0.05. ^#^ *p* < 0.0125

a compared to moderate differentiation

b compared to poor differentiation

c compared to high differentiation

### Distribution of TAMs was associated with EMT in Kazakh ESCC tissues

To investigate the role of TAMs in the tumour EMT process, the relationship between the distribution of TAMs and EMT-related protein was analysed. The density of interstitial TAM infiltrates positively correlated with vimentin expression (*p* < 0.05) and was not related with E-cadherin expression (*p* > 0.05). There was a negative correlation between the expression of E-cadherin and vimentin (*p* < 0.05) (Table 4). These results indicated that TAMs were positively associated with EMT in ESCC.

**Table 4:** Cross correlation analyses reveal strong relationships among density of TAM in tumor nest, tumor stroma and expression of E-cadherin and Vimentin in Kazakh ESCCs

Characteristics	TAM density in tumor nest *r / P*	TAM density in tumor stroma *r / P*	E-cadherin *r / P*
TAM density in tumor nest	1		
TAM density in tumor stroma	0.380 /<0.001*	1	
E-cadherin	0.066/0.517	−0.112/0.272	1
Vimentin	−0.166/0.102	0.319/0.001*	−0.619/< 0.001*

Partial correlation analysis method was employed to calculate the correlation coefficient of the corresponding two variables(eg. TAM in tumor nest and TAM in tumor stroma) with adjusting the other two variables (eg. expression of E-cadherin and expression of Vimentin). *p<0.05

### TAMs induce cancer cells to undergo EMT in ESCC

**Fig. 3:**
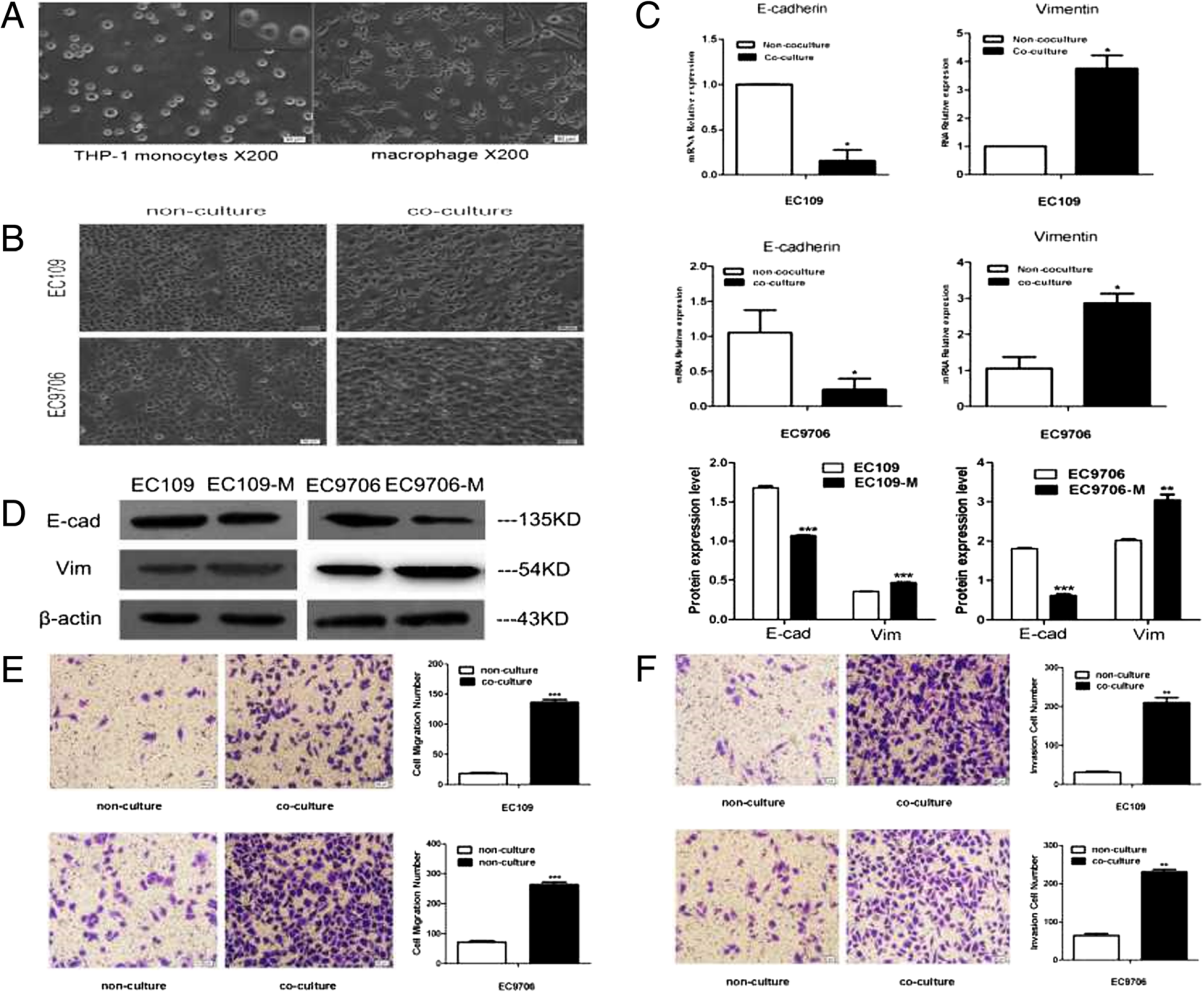
TAMs induce the cancer cells to undergo EMT. (**a**) PMA-treated THP-1 cells were differentiated into adherent macrophages. (**b**) EC109 and EC9706 cells presented typical cobblestone and epithelial-like appearances, respectively; with CM treatment, they were scattered and spindle shaped, and exhibited fibroblast-like appearances. The photographs were taken at 100x magnification. (**c**) The relative mRNA expression levels of E-cadherin and vimentin were determined by qPCR, respectively. The housekeeping gene GAPDH was used as the control. (**d**) The EMT-related proteins E-cadherin and vimentin were examined by western blotting, respectively; β-actin was used as a loading control. (**e**) and (**f**) Macrophages promote the migration and invasion of ESCC cells, respectively. * *P* < 0.05, ** *P* < 0.01, *** *P* < 0.001

THP-1 cells are usually selected as a model for studying human macrophage differentiation. In this study, suspended, round-shaped THP-1 cells were induced to adherent macrophages upon treatment with PMA for 36 h (Fig. 3a). To investigate whether TAMs could promote EMT of ESCC cells, we co-cultured THP-1-derived macrophages with ESCC cell lines (EC109 and EC9706) for 48 h and found that the morphology of ESCC cells transformed from round epithelial cells into loose, scattered, spindle-shaped mesenchymal cells, suggesting that EMT occurred in the presence of macrophages (Fig. 3b). To further confirm EMT, the pattern of gene expression was analysed by qPCR. mRNA expression of E-cadherin decreased, whereas that of vimentin increased in EC109 and EC9706 cells (*p* < 0.05) (Fig. 3c). Western blot analysis showed that the expression of E-cadherin decreased significantly, whereas that of vimentin increased significantly in the presence of TAMs (*p* < 0.05) (Fig. 3d). These results suggest that TAMs promoted EMT of ESCC cells, which were consistent with the results from in vivo tissue studies.

### TAMs enhance cancer cell migration and invasion in vitro

A transwell assay was used to investigate whether TAMs also influenced the migration and invasion ability of ESCC cells. The results in Fig. 3e and f showed that the migration and invasion ability of ESCC cells cultured in macrophage CM was significantly higher than that of cancer cells cultured in regular media (*p* < 0.05), suggesting that TAMs promoted the migration and invasion of ESCC.

## Discussion

TAMs are involved in tumour progression and are associated with poor prognosis in several cancers [21–24]. To our knowledge, EMT is one of the most important mechanisms of tumour invasion and metastasis [25]. However, the interaction between the TAMs and EMT in ESCC has not been elucidated clearly. Investigation of the underlying mechanisms may reveal new targets for EC treatment.

Our previous study shows that the number of M2 TAMs positively correlated with malignant phenotypes including lymph node metastasis and clinical stage progression of Kazakh ESCC [18]. On the basis of previous studies, we add some new Kazakh ESCC samples and found that CD163-positive TAMs were primarily located in the tumour stroma, and a few were also distributed in the tumour islet. The density of TAMs in the tumour islet and stroma was significantly higher than that in the corresponding CAN tissues. Our results were similar to those reported in gastric cancer [26], oral carcinoma [27], and endometrial adenocarcinoma [28], indicating that TAMs play a crucial role in the promotion of oesophageal cancer invasion and metastasis. EMT is required for tumour invasion and metastasis. To investigate whether TAMs were involved in EMT of cancer cells to promote the invasion and metastasis of ESCCs, we analysed the expression of an epithelial marker (E-cadherin) and mesenchymal marker (vimentin) in ESCC samples. We found that E-cadherin expression negatively correlated with the number of TAMs and was significantly downregulated in ESCC. However, vimentin expression positively correlated with number of TAMs and was significantly upregulated in ESCC. Furthermore, we found that the expression of E-cadherin was significantly down-regulated in ESCCs compared with CAN tissues. Low expression of E-cadherin was closely related to the depth of invasion, clinical stage, lymph node metastasis and vascular invasion of ESCCs. Whereas the expression of Vimentin was significantly up-regulated in ESCCs, and the overexpression of Vimentin was closely related to the depth of invasion, clinical stage, lymph node metastasis and vascular invasion of ESCCs. Through the analysis of Partail correlation, found that M2 TAMs in tumour stroma were positively correlated with Vimentin, but negatively correlated with E-cadherin in ESCCs, suggesting that the cancer cells may be reprogrammed by the TAMs and transformed into cancer cells with more mesenchymal-like properties [29, 30].

In vitro experiments showed that TAMs not only promote the transformation of ESCC epithelial cells into spindle-shaped mesenchymal cells, but also affect the expression of EMT cell molecular markers (E-cadherin and vimentin) at the mRNA and protein levels. The migration and invasion of ESCC cells were significantly increased, suggesting that TAMs have important biological functions in promoting EMT, invasion, and metastasis of ESCC cells. During this process, TAMs may produce certain cytokines by autocrine and paracrine factors that might play an important role in EMT of ESCC cells. However, the role of these factors was not explored in this present study. Therefore, in future we plan to further examine the changes in cytokine levels in the co-culture system to explore the specific mechanisms of TAMs in promoting EMT in ESCCs.

## Conclusions

In summary, here, we report the relationship between TAM and EMT in ESCC. TAMs are closely related to cancer cell EMT and are involved in the invasion and metastasis of Kazakh ESCC. Our study emphasizes the importance of the mutual interaction between the TAMs and EMT of cancer cells in tumour progression and provides new ideas for the treatment and prevention of metastasis and recurrence of ESCC.

## Data Availability

Data and materials will be shared.

## Abbreviations

CANs: Cancer adjacent normal tissues
CM: Conditioned medium
EC: Oesophageal carcinoma
EMT: Epithelial to mesenchymal transition
ESCC: Oesophageal squamous cell carcinoma
IFN-γ: Interferon gamma
IL-10: Interleukin-10
IL-12: Interleukin-12
IL-13: Interleukin-13
IL-4: Interleukin-4
LPS: Lipopolysaccharide
PMA: Phorbol myristate acetate
TAM: Tumour-associated macrophage
